# Assessment and Solutions to Food Waste at Congress Events: A Perspective of the MagNuS Project

**DOI:** 10.3390/foods13020181

**Published:** 2024-01-05

**Authors:** Maria-Angeles Fernandez-Zamudio, Inmaculada Zarzo, Tatiana Pina, Jose M. Soriano, Nadia San Onofre

**Affiliations:** 1 Centro para el Desarrollo de la Agricultura Sostenible, Instituto Valenciano de Investigaciones Agrarias, 46113 Moncada, Valencia, Spain; 2 University Clinic of Nutrition, Physical Activity and Physiotherapy, Lluís Alcanyís Foundation-University of Valencia, 46020 Valencia, Valencia, Spain; farmarzar@hotmail.com; 3 Department of Experimental and Social Sciences Education, University of Valencia, 46022 Valencia, Valencia, Spain; tatiana.pina@uv.es; 4 Food & Health Lab, Institute of Materials Science, University of Valencia, 46980 Paterna, Valencia, Spain; jose.soriano@uv.es (J.M.S.); sanonofre.nadia@gmail.com (N.S.O.); 5 Joint Research Unit on Endocrinology, Nutrition and Clinical Dietetics, Health Research Institute La Fe-University of Valencia, 46026 Valencia, Valencia, Spain; 6 Department of Community Nursing, Preventive Medicine and Public Health and History of Science, University of Alicante, 03690 San Vicente del Raspeig, Alicante, Spain

**Keywords:** food waste, quantification, MICE, nutritional value, sustainability

## Abstract

Addressing global food waste is a formidable challenge, requiring comprehensive efforts across the food supply chain, particularly in sectors prone to waste like HoReCa (Hotel, Restaurant, and Catering). In MICE (Meetings, Incentives, Conventions, and Exhibitions) tourism, where gastronomy is a major attraction, overlooked meal services during working meetings contribute significantly to food waste. The MagNuS (Magnitude, Nutritional value, and Sustainability) project aims to assess and address food waste during conference events at the Valencia Conference Center in Spain. This study quantifies waste, categorizes it by food groups, estimates the potential number of individuals that could be fed with discarded food, and assesses energy and nutritional values. Across three events, 104.4 kg of food waste was documented, with cereals having the highest wastage, followed by legumes, fish, and others. Acknowledging potential underestimation due to reliance on cooked values, this study suggests using residues for vermicomposting or composting as sustainable waste management alternatives. These findings have implications for future initiatives, advocating diverse strategies to minimize food waste during congress events, aligning with the Sustainable Development Goals. The MagNuS project contributes valuable insights to sustainable practices in MICE tourism, informing policies and operational decisions to reduce the environmental impact of food waste.

## 1. Introduction

**Figure 1:**
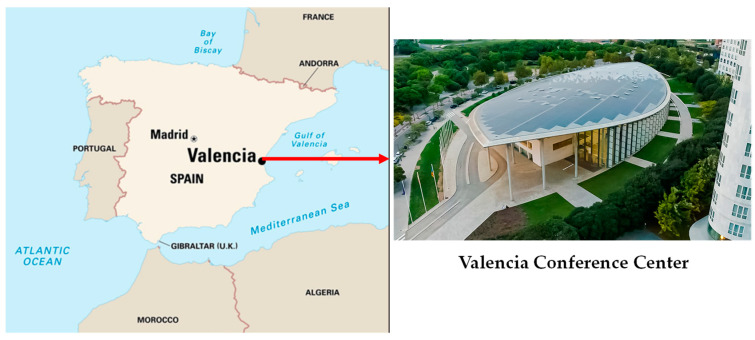
Location of Valencia Conference Center.

In 2022, the global tourism industry made a remarkable recovery with 969.40 million travelers, doubling the previous year’s figures. However, this still represents a 34% decline from 2019 levels (1463.68 million) as estimated by the World Tourism Organization (UNWTO) [1]. Europe led the way with 61% of global tourists, followed by America (16%) and Asia (7%). Within Europe, Spain, France, Turkey, Germany, and Greece were among the most popular destinations. Tourism significantly contributes to job creation, with Spain being a notable example of a country benefiting from tourism-related employment. Shifting the focus to the noteworthy case of Spain, statistics reveal that in 2022, this country received 71.6 million tourists, which reflected 129.5% more compared to 2021, with the Valencian Community being the fourth autonomous region, behind the Canary Islands, Catalonia, and Andalusia, in terms of tourist reception [2]. The Valencian Community is working on the concept and the connection of tourism focused on five tourism niches: spas/health/wellness [3], culinary/food/wine [4], sports [5], creative/cultural [6], and MICE (Meetings, Incentives, Conventions, and Exhibitions) [7] tourism, with great success to date. It is integrating sustainability principles into each of these five niche concepts [8], aligned with the Sustainable Development Goals (SDGs) [9]. MICE tourism, as a sector of the tourism industry, holds notable economic relevance, which has been steadily growing in recent years [10]. In fact, the global MICE market was valued at USD 876.42 billion in 2022 [11] and Spain is ranked as the fourth country in the world in terms of international meetings held [12]. In the Valencian Community (Spain), the greatest business convention center is known as the Valencia Conference Center (Figure 1). This distinguished venue has been awarded as the World’s Best Convention Center twice, in 2018 and 2010, representing the most prestigious international award from the International Association of Convention Centers, which recognizes excellence in management, facilities, equipment, services, accessibility, and accommodation capacity. Furthermore, the center was also awarded, in 2001, with the first annual prize from the Spanish Federation of Professional Convention Organizers for the quality of its service and facilities provided to the city. The building is a landmark architectural work by the celebrated architect Norman Foster. In 1999, it was awarded Best European Building by the Royal Institute of British Architects, a year after its opening [13]. The strategic and operational planning of the Valencia Conference Center has been focused, since its origins, on sustainability, seeking to guarantee that the interaction between the building and the events held there helps preserve nature [14].

In that context, the aims of this work were to carry out the first study measuring food waste at congress events, estimate its energy nutritional value, and propose alternatives to minimize or eliminate that waste. The structure of this paper is as follows. Following the introduction to the topic, the literature review is expounded as a theoretical foundation for this research applied in the restaurant of the Valencia Conference Center. The third section encompasses the materials and methods employed in this study, while the fourth section delves into the presentation of results derived from these methodologies. The disclosed results are further detailed in conjunction with a discussion, highlighting our assessment and proposed solutions to food waste in the Valencia Conference Center. The conclusion section addresses the theoretical and practical implications of the application of this focus on food waste, along with the study’s limitations, proposals for future research, and implications.

## 2. Literature Review and Theoretical Basis

### 2.1. Literature Review

Conferences and business meetings are typically held in convention centers and are characterized by a blend of work and leisure, with mealtime being a key aspect. Meals during these events serve as opportunities to enrich social relationships among attendees [15]. Such events often gather many people, necessitating complex logistics, including food preparation and distribution to meet attendees’ needs [16]. In this logistic process, sustainability has become an essential requirement, for both organizers and all stakeholders in the MICE industry [17], including the venues hosting these events, which are recognizing the need to adopt greener practices [18]. In this regard, integrating the SDGs into the planning and execution of these events should promote more responsible practices [19]. Although there are numerous aspects to consider for achieving event sustainability, food is one of the elements that requires special attention due to its significance [20]. In fact, as highlighted in the study of Read and Muth [21], food is one of the aspects with the most requirements when organizing a conference, and, simultaneously, it can be a significant source of complaints from participants. Given that dissatisfaction with food has a significant impact on any HoReCa (Hotel, Restaurant, and Catering) business, extensive research has been conducted to understand both diners’ [22] and HoReCa channel managers’ [23] perspectives. HoReCa is a rather complex sector that includes several divergent activities and rather diverse consumers [24,25,26]. One crucial indicator for identifying potential shortcomings in the served meals is the volume of food waste generated on the plate. In the EU in 2021, the total measured food waste exceeded 58 million tons of fresh mass (approximately 131 kg per person), with an estimated market value of EUR 132 billion [27]. However, the impact of food waste extends beyond mere economic implications, encompassing broader environmental concerns. In 2020, it was estimated that food waste was responsible for 252 Mt of CO_2_ emissions [28], constituting about 16% of the total greenhouse gas emissions from the EU food system [29]. The discarded food also influences our use of resources, such as freshwater, cropland, and fertilizers [30]. Additionally, food waste adversely affects the nutritional health of millions of people. In fact, the 2023 report on the State of Food Security and Nutrition in the World [31] discloses that in 2022, up to 783 million people worldwide experienced hunger, and more than 3.1 billion people were unable to afford a healthy diet in 2021. It is, therefore, imperative to establish strategies that reduce food waste and promote food sustainability in this industry.

This heightened awareness about food waste began when the FAO released its first comprehensive study presenting global figures in 2011 [32] and followed up in 2013 with a global study on the social, environmental, and economic impacts of this issue [33]. Food wastage spans the entire food chain. Within the supply chain, from harvesting to processing, approximately 13.8% of food is wasted [34]. A considerable portion of this waste is generated in the food services industry. In developed countries, waste tends to concentrate in the final consumption phase, where 12% of food waste is generated in the food service sector, including restaurants, bars, bistros, fast food chains, catering, etc. [35]. This sector ranks as the third-largest generator of food waste in Europe, following households (53%) and food manufacturing and processing enterprises (19%) [35]. In the UK, hospitality businesses generate around 0.9 million tons of food waste annually, accounting for about 10% of the national food waste stream [36]. In selected European countries, the waste in the hospitality sector reaches, on average, 20% of meals [37]. Managing the issue of food waste in the hospitality sector is a matter of concern and scientific analysis [23,38]. Studies aim to assess the ecological footprint of this industry [39] and often conclude that preventing and reducing waste figures usually translates into a double gain for both consumers and businesses [21]. This also presents an excellent opportunity for hospitality businesses to enhance their reputational image, as they find in this challenge a chance to improve their public image [40]. Furthermore, a non-academic study, drawing data from 114 restaurants, has found that every 1 USD invested in food waste reduction generates an average of 7 USD in savings over a three-year time frame [41]. 

Undoubtedly, the first step in addressing the challenge of reducing food waste is measuring it, since these figures are crucial to raising awareness of the problem and seeking solutions [36,42], always from a sustainability perspective [19]. This is reflected in target 12.3, which specifically addresses these statistics and states, “By 2030, halve per capita global food waste at the retail and consumer levels and reduce food losses along production and supply chains, including post-harvest losses” [43]. If we aim to halve waste, we need to know the starting figures. To advance in obtaining reference figures and in defining standardized methods for quantifying food waste, the European Union issued Commission Delegated Decision (EU) 2019/1597 on 3 May 2019 [44], which outlines a common methodology and minimum quality requirements for uniform measurement. In the case of restaurants and food services, this Decision proposes direct measurements (weighing or volumetric assessment), waste composition analysis, and/or counting/scanning to establish the amount of food waste. Accordingly, validated measurement methods adapted to all stages of the food chain are of growing interest. These include economic instruments, among others, to reduce fossil fuels and animal-derived products [45,46]; some taking into account geographical variations [47]; the use of new tools for structured assessment and a more informed selection of waste management methodologies for each category of food waste [48]; and application of digitization and AI to avoid harvest losses [49].

### 2.2. Theoretical Basis

Three research question are posed here, and in the literature, only one study [50] was conducted with these three investigative items, in hospital food waste; to the best of our knowledge, they have not been applied in MICE. The interconnection between food waste, nutrition and health, and sustainability in tourism and the HoReCa sector underscores the need to comprehensively address these challenges to create a more equitable and environmentally respectful world. Accordingly, a new innovative project on food waste, MagNuS (Magnitude, Nutritional value, and Sustainability), has been implemented in the restaurant of the Valencia Conference Center. Based on this, the present study posed the following research questions:

RQ1. What factors should be considered for systematically measuring waste generated in a specialized collective catering service for MICE (Meetings, Incentives, Conferences, and Exhibitions) events? The first research question is focused on identifying the key factors that need to be taken into account when measuring waste in a specialized catering service for events, particularly those related to tourism MICE. The question emphasizes the systematic nature of the measurement, suggesting the need for a structured and comprehensive approach to waste assessment. 

RQ2. What is the energy and nutritional estimation of the waste generated during events, which may potentially be utilized through alternative means for waste reduction or elimination at the Valencia Conference Center? This goal is to explore ways in which this waste can be repurposed or utilized through alternative channels to reduce or eliminate waste at the venue. The question reflects an interest in understanding the potential value and utility of the waste in terms of both energy and nutritional components. 

RQ3. What proposals can be made to enhance the sustainability of an emblematic site such as the Valencia Conference Center? This seeks to identify proposals or suggestions for strengthening the sustainability of a prominent venue, specifically the Valencia Conference Center. This question implies a focus on sustainability initiatives and strategies tailored to the context of a specific landmark. The term “emblematic site” suggests that the venue holds significance, and the question aims to explore ways to make it more environmentally and socially sustainable in terms of food waste by applying eco-friendly practices.

**Figure 2:**
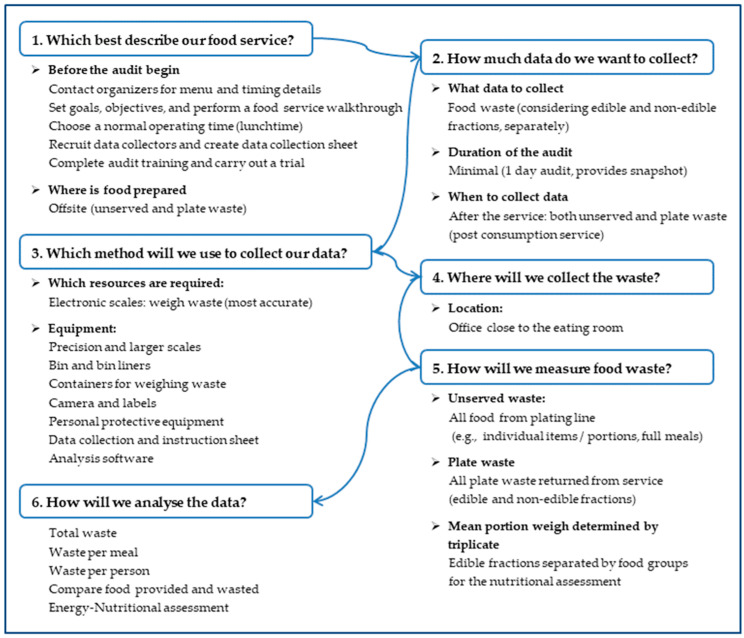
Consensus tool developed by the Department of Nutrition, Dietetics, and Food at Monash University in Australia [51] and adapted to this study.

In summary, this study develops a conceptual model based on the framework proposed by Cook et al. [51] at Monash University. This framework promotes the adoption of a comprehensive diagram, similar to an audit, where stages are systematically fulfilled to define the type of waste to be measured and other critical considerations for quantifying waste in collective catering. These stages involve posing and partially answering questions, collectively contributing to a systematic and efficient quantification process. The original diagram proposed in Cook et al. [51] was adapted to the specific circumstances of a venue like the Valencia Conference Center. In our case, we prioritized six key aspects: (i) What best describes our food service? Data from both pre-measurement information provided by the catering company and post-measurement information specifically focused on food discarded by diners (post-consumption service) are used; (ii) How much data do we want to collect? Details of the measurement are provided, distinguishing each menu item, considering only the edible fraction as waste (usually the majority of what is served), measuring during a main congress meal, and collecting quantifiable leftovers after the meal service has ended; (iii) What method will we use to collect data? Defining the sample scope, in our case, recording all generated leftovers, using precision scales to measure different weights; (iv) Where will we collect the waste? Opting to place the measurement area near the station used by waiters for general collection and dish cleaning; (v) How will we measure food waste? Identifying additional measurement details and the various quantitative variables to be recorded; and (vi) How will we analyze the data? Determining total volumes of generated waste, waste per type of dish or per capita, as well as other variables of interest, thereby obtaining a large amount of data that also contribute to completing the nutritional assessment. To provide a clearer visualization of the entire process and the aspects contributing to quantification, Figure 2 has been created.

## 3. Materials and Methods

### 3.1. Characteristics of the Studied Congress Events

Although the types of food services offered by the catering company at Valencia Conference Center (Gourmet Catering & Events) are diverse, in practice, most of the events are executed with menus that follow the format of the three congresses analyzed here, which are reflected in Table 1. In the present study, a total of 1020 diners participated in these three events where food waste was measured. This catering service is of the full-service restaurant catering type, including meal preparation, decorations, and clean-up following the event.

In the present research, a cross-sectional study, all participants of the three congresses ate independently and they had different professions but the topics of the three congresses were related to health fields. 

### 3.2. Quantification of the Magnitude of Food Waste

For quantification purposes, identified by the initials “Mag” in the acronym MagNuS project, a protocol was generated for these types of events based on the study conducted by Cook et al. [51] and adapted for implementation at the Valencia Conference Center. The quantification of food waste at events held at the Valencia Congress Center was carried out following the steps specified in Figure 2. In practice, any measurement protocol is realized by determining a set of numerical variables that should be quantified for a thorough understanding of the food waste situation in a MICE tourism establishment. These variables are, therefore, the data that constitute the results of the measurement part. These variables have been defined and summarized in Table 2. It is worth noting that we applied precision scales to measure different fractions of the dishes and other larger-capacity scales (KERN FCB 30K1, Kern & Sohn GmbH, Balingen, Germany) to measure the volume of discarded organic waste. Mean portion weight, of the served and discarded food, was determined in triplicate using a calibrated kitchen scale with an accuracy of 1 g. 

Among all the approaches that can be used to gather data on the level of food waste an organization generates, the most rigorous and accurate is on-site weighing, as it can capture the food waste data at the point of generation before it is mixed with other waste streams. This involves weighing the food waste at the source of generation, using scales or bins with embedded sensors. This method can provide accurate and detailed data on the amount, type, and source of food waste, as well as the potential causes and solutions. However, this method can also be costly, time-consuming, and require staff training and engagement [52,53]. In alignment with the division proposed by several authors [23,54], the extent of food waste generated in the pre-kitchen (storage) and in-kitchen (preparation, serving, and leftovers) phases is largely influenced by company business practices and supplier engagement. In contrast, food waste produced in the post-kitchen phase is predominantly influenced by consumer behavior. Notably, plate waste (i.e., post-kitchen) ranks as the second leading source of waste within the HoReCa sector.

The calculation of food waste was determined by a set of quantitative variables collected in Table 2. The figures are presented in both absolute values, such as the total volumes of discarded food at an event, and in the form of more relative values, for example, related to waste by type of dish (or portion) or per capita. The latter allows for comparisons of results obtained between different measured events. Among all the calculated variables, those that best demonstrate the impact of the measurement are presented in Table 3 of this study: the magnitude of overall waste (in grams), waste per person and per type of dish (expressed in grams or as a percentage), as well as the estimated number of portions wasted for each type of dish.

### 3.3. Assessment of Nutritional Content and Sustainability 

The nutritional content, represented by the initials “Nu” in the acronym MagNuS, was measured directly at the establishment. Meals, served and discarded at the congress events, were weighed ingredient to ingredient in each meal. These values were introduced in a validated nutritional software package (DIAL Version 3.0.0.5; Alce Ingeniería SA, Madrid, Spain), which obtains energy, fiber, and macro- and micro-nutrients from the food composition table [55]. Finally, we explored different viewpoints aimed at reducing or eliminating, in the future, the food waste generated at congress events, aligning with the “S” in the acronym MagNuS. Studied meals were grouped in the food groups established by the EAT-Lancet Planetary Health Diet [56].

### 3.4. Statistical Analysis 

To analyze the data, a descriptive statistical analysis was conducted. IBM^®^ SPSS^®^ Statistics version 27 software (IBM Corp., Armonk, New York, NY, USA) was used for statistical analysis. Quantitative variables were described with their mean and standard deviation.

## 4. Results

### 4.1. Quantification of the Magnitude of Food Waste

The measurement carried out at three different congresses was focused on determining the post-consumer food waste, i.e., the volume of food left over as either leftovers or full portions that were either not served, despite being scheduled for such services, or were not eaten at all. Table 3 shows meals served during the three congress events, as well as the quantity and proportion of waste generated.

At the first congress event, 24.1 kg of food was wasted; at the second event, the amount of waste increased to 49.6 kg; and at the third congress, 30.7 kg of waste was recorded. Altogether, this adds up to a total of 104.4 kg of food waste. Regarding wasted plates, or portions in the first measurement, 104 plates were wasted, which was 5.9% of the total plates served. In the second measurement, the number of wasted plates reached 1712, representing 27.2% of the plates served. In the third measurement, 927 plates were discarded, corresponding to 15.6% of the plates served. Analyzing these figures on a per-diner basis, it can be observed that in the first measurement, there was a food waste of 89.2 g per person, representing 14.21% of the quantity served to each individual. In the second measurement, a waste of 123.9 g per person was generated, equivalent to 17.36% of the portion served to each diner. In the third measurement, the waste figure was 87.6 g per person, implying 12.58% of the quantity served per individual.

### 4.2. Assessment of Nutritional Content and Sustainability

According to food groups classified with the EAT-Lancet Planetary Health Diet [56], Table 4 shows the food waste at the three congress events. None of the events used nuts or eggs as primary ingredients in the dishes served, so no food wastage was generated for these items, as shown in Table 4. However, food waste was observed at one congress event for the legumes, added fats, and ultra-processed foods categories, and at two events for the fruits, tubers, and red meat group. The remaining food groups showed waste at all congress events. According to these values, it can be observed that the higher values of food waste were associated with cereals, followed by legumes, fish, red meat, dairy, tubers, vegetables, fruit, ultra-processed foods, white meat, and finally, added fats. 

The quantity of cooked food compared to its raw form implies that the value of the cooked food is lower than that of the raw form. The number of people who could have been fed with the food waste/food group was calculated (undemonstrated data); however, the values would reflect an underestimation compared to the raw food. If we make an approximation, considering the reference value as cooked, in the case of cereal residues alone, they could feed approximately 74 people, or even 130 people with fish.

In Table 5, the energy nutritional assessment of food waste generated at each congress is presented. The interest in the nutritional energy assessment of these residues lies in their subsequent use in two senses. First, considering the reference dietary intakes [57], these residues could meet the dietary reference intakes for 150 people/day in terms of energy. Second, the nutrients contained in the food residues are valuable for earthworms or microorganisms if the studied food service wants to use them for vermicomposting or composting, respectively.

Finally, the results were focused on sustainability, corresponding to the initial “S” in the acronym MagNuS project. This project has identified four steps that could be taken to achieve the sustainability goal set out in the technical guide about sustainability in the Valencia Conference Center, as follows: (i) that the catering company uses of the Too Good To Go app to help reduce food waste and CO_2_ emissions and to allow the general public to access quality products at an affordable price [58], (ii) the Center could raise awareness among its events’ attendees with a mobile app called MySusCof, thus applying sustainability education to reduce the food waste of consumers [59], (iii) the leftover food could be used in charity canteens in Valencia by Foodration4all [60], and (iv) the Center could create compost from the food waste [61] and use it in the gardens on-site.

## 5. Discussion

**Figure 3:**
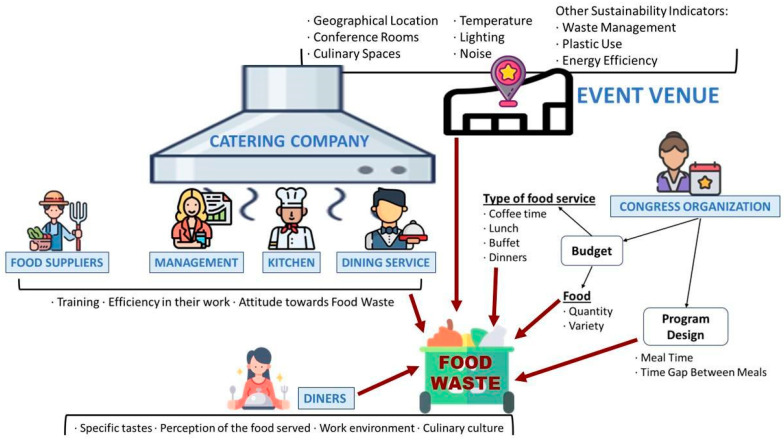
Key factors used for Valencia Conference Center to calculate the food waste at the congress events.

The answers to the questions raised in the Theoretical Basis subsection are as follows. First, Figure 3 has been developed to answer RQ1; it summarizes the key factors that influence the potential food waste originating from an event, since a greater or smaller proportion of the food served is wasted depending on multiple factors and people. 

The venue itself, where the congress is held, plays a crucial role. Factors such as geographic location, temperature, lighting, and noise within the facilities can influence how comfortably attendees dine, ultimately impacting potential waste. Another vital agent is the catering company, responsible for selecting and liaising with food suppliers, as well as organizing the cooking and food service in dining areas or gastronomic spaces. The various individuals involved in these preparations influence the quality of the food served and how attendees are catered to. Therefore, their training, dedication, and attitude toward food waste condition the volume of wasted food. The organizing personnel of the congress also play a pivotal role, as they have the authority to plan and decide the time and budget allocated to the catering services, choose the type of service, the menus, and consequently, the quantity and variety of food served during the event. Lastly, the most decisive factor is the one generating waste on-site, the congress participants or diners. Their personal tastes, perception of the food served, workplace environment, and culinary culture influence their attitude toward food, resulting in more or less food waste during the event days.

It is important to measure all data that are collected, and in this study, 1020 diners at the three congress events were investigated based on a case study of the Valencia Conference Center. The following group of issues are proposed to be considered if food waste is to be measured at other similar events:

(i) Nature of the event. Each type of event is different, not only in its audience but also in the catering service setup. It is essential to identify the gastronomic service schedule and choose the moment for measurement. For instance, the amount and type of food served during a coffee break or the time between the coffee break and the main meal is determinant. Understanding various details, such as the contracted services (which are highly personalized and tailored to the client’s demands), is crucial. In essence, understanding the initial event setup is necessary as it contextualizes the impact of the waste figures ultimately determined;
(ii) Number of attendees. Congress-type events typically gather a large number of people. The catering service will significantly change based on the number of attendees. The results are proportional to the number of people who have eaten, so the waste generated must be calculated on a per capita basis in order to make a comparison between different types of events. But one recommendation is that until proficiency in measurements is achieved, it is advisable not to start with extremely large events, as the measurement difficulty also increases with the event’s size;
(iii) Contracted catering service. Waste figures are often clearly influenced by the quantity and type of food contracted. Generally, the diners do not make the choice; it is made by the congress organizing committee or even a travel agency collaborating with the organization. This decision significantly affects what and how much food and beverages are served and is largely motivated by the available budget. Hence, there might be congresses where the food is abundant and oversupplied, while others may seem insufficient for all attendees;
(iv) Type of service. The method of serving food in the dining area or assigned space can vary significantly. Attendees might sit at tables or stand, and sometimes options are alternated because attendees choose where to eat, such as in a garden with both seated and standing options;
(v) Spaces for food waste measurement. An essential operational aspect is the available space for quantifying food waste. This varies at each event, depending on the physical space, which, in turn, depends on the type of food served, the number of attendees, etc. If space is limited and the service pace is very fast, the measurement task becomes complicated since there might not be enough time to weigh all the waste separately. Throughout, the priority should be to disturb the food service flow and plate removal and cleaning as little as possible;
(vi) Personnel for measurement. The number of people involved in the measurement task is also crucial. If insufficient, the measurement will be incomplete. It cannot be too many people; it should be the right number for the available measurement space and the pace at which plates come in from the dining area. Regardless of the number, it matters that these individuals are trained in the measurement task. They must clearly know what to record and conduct the weighing rigorously, noting all details that could influence the collected data.

Furthermore, food waste should be collected in the same catering service, assessed in situ in this place, and the data analyzed immediately. In the end, the following question arises: What can we do or how can we act to prevent and reduce the food waste that occurs at this type of event? Of course, it seems a priority to fully involve the company in charge of catering, as there are many actions it can take in the cooking and serving phases of the food. It is also important to involve those who organize the congresses, who are ultimately the clients who determine the characteristics of the food served. Many actions can be encouraged jointly, and this is also reflected in the literature that is gradually being published in this area. According to Vizzoto et al. [62], companies should focus on constantly revising the dishes offered on the menu, reducing overcooking, exploring creative repurposing, considering donations to staff/charity organizations, offering options for ordering smaller portions, and implementing marketing actions. For waste reduction, companies typically employ several actions [41], including measuring, involving staff, cutting overproduction, reassessing inventory and purchasing practices, and repurposing excess food. Additionally, food waste reduction may benefit from management practices commonly used in manufacturing companies. Gladysz et al. [63] examined the application of “lean management practices”, that is, producing more with less. To fully understand best practices in HoReCa, companies aiming to reduce food waste should consider the impact of suppliers and consumer behavior. Efforts to reduce food waste should concentrate on innovating menu planning, purchasing, and food preparation processes [64]. Local suppliers, in particular, often demonstrate more flexibility and responsiveness to changes in demand. This is especially advantageous in the tourism sector, where collaboration with local suppliers offers additional benefits [65]. Understanding customer needs contributes to reducing food waste. For instance, extending lunch breaks led to a reduction in plate leftovers [66].

On the other hand, the interconnection between food waste, its nutritional value, and the presence of hunger is a multidisciplinary and global issue that requires deep understanding and effective action helping to respond to RQ2. Addressing this dilemma can have a significant impact on the fight against hunger and the promotion of a more equitable distribution of food resources worldwide. In this context, it is essential not only to reduce food waste but also to recognize that discarded food, if managed properly, can have remarkable nutritional value and provide assistance to many individuals in need. As reflected in Table 5, the food waste from conferences has the potential to make a significant contribution to the nutrition of vulnerable individuals. This underscores the importance of finding innovative and effective solutions to redirect unconsumed food to those who need it the most. Collaboration between governments, charitable organizations, food industries, and society as a whole is crucial for addressing this issue comprehensively. By doing so, we can work towards a future where the paradox of waste and hunger is replaced by a fairer balance in the distribution of food resources, promoting nutrition and food security worldwide.

Last, the technical guide about sustainability in the Valencia Conference Center has been studied, and in response to RQ3, appropriate steps sourced from the scientific literature. The Too Good To Go app is easy to use, clear, to-the-point, and helps everyone to access quality products at an affordable price [67]. However, Mathisen and Johansen [68] carried out a study, in a small sample of students (*n* = 6), with a mean age of 24.7, where this app did not result in any measurable effects, that is, no change in food waste, healthy eating, or personal food expenses. Evans [69] demonstrated how discrepancies in food procurement and consumption led to the generation of food waste. Nonetheless, preventing and reducing waste is a major challenge facing society; achieving the combination of food waste utilization and healthy eating requires comprehensive health education for the public. It is not about partial strategies but about launching collective projects that are both multi- and interdisciplinary. Although there is an app (MySusCof) [59] focused on educating people about the sustainability potential of food waste, there is currently no app that enables simultaneous education on sustainability (highlighting the importance of utilizing food waste), nutrition (promoting healthy eating), and culinary skills (facilitating the creation of recipes with the foods available to consumers, automatically). The use of food waste for charity canteens from Valencia with Foodration4all [60] is proposed, where the charity uses a blockchain application to purchase cards to obtain food/reduce food waste [70]. Lastly, the production of compost from food waste and its use for houseplants and gardens has been explored for university campuses [71], domestic households [72], and hospitals [73], among others, but so far, it has not been implemented in conference centers. 

## 6. Conclusions

The increasing relevance of MICE tourism brings attention to the significant issue of food waste, particularly at large-scale events associated with gastronomic moments. Despite the lack of baseline figures in the sector, addressing food waste requires a proactive approach involving awareness, consciousness, and the collection of detailed data, as specified in Delegated Decision (EU) 2019/1597 [44], preferably in situ, and preferably taking on board findings from various references that offer valid conclusions and proposals after exploring the situation of the problem. The Valencia Conference Center has taken a crucial step by measuring food waste at three different events, providing valuable insights into the variability of waste generation. These measurements serve as a foundation for learning lessons and making informed decisions to address the issue. The magnitude of wasted food necessitates immediate action, and the causes involve various actors, with event organizers playing a key role in budget allocation, food planning, and delivery. Factors such as the serving method, food quality, and the catering company’s role also influence food waste. By measuring food waste, a powerful message is communicated to those handling food, fostering awareness and prompting changes toward more mindful practices. The MagNuS project emerges as an initiative that not only quantifies discarded food but also raises awareness and motivates positive changes. It facilitates continuous learning and collaboration among stakeholders, positioning them as part of both the problem and the solution. The project’s measurement protocols can be adopted by other HoReCa companies, ultimately contributing to economic and environmental assessments of food waste. MagNuS extends beyond quantitative figures, addressing the socially unacceptable nature of food waste from an ethical standpoint. Recognizing the nutritional value of discarded food highlights its potential impact in nourishing others, emphasizing the importance of responsible practices. In essence, MagNuS, true to its name, continues to grow, expanding its focus to address additional issues like finding alternative outlets for usable food and utilizing leftovers. Tackling food waste emerges as a multifaceted and essential initiative for promoting the global sustainability of emblematic sites like the Valencia Conference Center. Based on the data obtained in this study regarding the scale of food waste generated, the future outlook will be focused on working with such waste and achieving its minimization, or even complete elimination of these unconsumed food items. Two limitations have been detected in this study, namely, that the sample comprised a high proportion of females vs. males, a common occurrence at congresses in Valencia, and the sample was concentrated on users from Spain and other countries of Europe, making it non-representative of participants from other parts of the world.

## Figures and Tables

**Table 1:** Types of food service at the congress events held at the Valencia Conference Center.

	Congress Event		
	1	2	3
**Type of menu**	Seated and featured a conventional menu	Accommodated and included a standing food service with a tapas menu	Hosted and offered a standing food service with a tapas menu
**Number of diners**	270	400	350
**Congress event date**	5 May 2023	25 May 2023	1 June 2023
**Geographic location**	Valencia (Spain)	Valencia (Spain)	Valencia (Spain)
**Temperature (°C) ^a^**	18.3–20.1	19.4–21.0	19.8–21.5
**Lighting (Lux) ^a^**	500–610	520–630	550–650
**Noise (dB) ^a^**	58–65	62–75	59–68

^a^ Temperature, lighting, and noise data, as minimum and maximum values, at the location where meals were served in the Valencia Conference Center.

**Table 2:** List of quantitative variables used in the determination of food waste.

**Initial information, taken from the catering company prior to the catering service**	
Number of diners or number of servings	Number of catering services that are contracted by the event organizers based on the number of people they estimate will attend as diners.
Number of dishes per person	Number of dishes that compose the menu served. For example, the number of different tapas served at Event 2 and Event 3.
**Variables determined prior to the collection of food waste in the dining room, and which serve as a basis for the nutritional characterization of the food served**	
Weight per portion	Amount of food (in grams) per individual portion of contracted and served food of each dish at each conference, so the portion size tends to vary according to the specificities and requirements requested for each event.
Weight of main ingredients	Grams that the main ingredients of each portion weigh separately.
Inedible fraction percentage	For dishes containing inedible items, the percentage of the weight of the portion that represents this fraction.
**Variables obtained after the consumption of the diners in the dining room, and which reflect the results of the food waste figures of the event**	
Total wastage per portion	Volume of food (in grams) collected from each dish after service for all diners.
Waste per capita and per dish	Volume of waste from each dish (in grams) divided by the number of diners.
Number of wasted portions per dish	The equivalence derived by dividing the total volume of waste generated for each type of dish by the weight of each portion.
Number of portions not served in the dining room	Portions that were prepared but do not even make it out of the kitchen because there is no demand for them. They are potentially usable.
Number of portions not consumed	Portions that go to the dining room, but are collected intact because they are not consumed. If the food legislation allows it, they could potentially be usable.
Percentage of wastage per portion and per capita	A percentage obtained by multiplying the waste per capita and per portion by 100 and dividing that by the weight per portion.
Total volume of wastage for the event	The sum of all waste generated in the service, in grams. It is obtained from the sum of the volumes of food collected as wasted food remains, in each of the dishes, plus the weight added for the portions not served in the dining room, or the portions not consumed in the dining room.

**Table 3:** Dishes served at the studied three congress events, along with magnitude and estimated portions generated as waste.

Dish	Magnitude Generated Waste (g)	Estimated Food Waste (in Grams/Portions *)			Estimated Food Waste (in Grams/Percentage per Portion) per Capita		
		Event 1 ^a^ (*n* = 270)	Event 2 ^a^ (*n* = 400)	Event 3 ^a^ (*n* = 350)	Event 1 ^a^ (*n* = 270)	Event 2 ^a^ (*n* = 400)	Event 3 ^a^ (*n* = 350)
Salad	3823	2935/14	888/28	N/A ^e^	10.87/5.0	2.22/7.09	N/A
Valencian paella ^b^	6421	6421/39	N/A	N/A	23.78/14.6	N/A	N/A
Vegetable paella	16,785	N/A	6654/34	10,131/59	N/A	16.64/8.43	28.95/16.87
Fideua ^c^	14,362	3854/32	4773/24	5735/34	14.27/11.7	11.93/6.1	16.39/9.62
Natural yogurt with tangerine ice cream	2451	2451/19	N/A	N/A	9.08/7.1	N/A	N/A
Bread	8425	8425/176	N/A	N/A	31.20/65.0	N/A	N/A
Fine zucchini coca with black olives	279	N/A	210/10	69/2	N/A	0.53/2.54	0.20/0.58
Cheese and quince skewer	65	N/A	37/6	28/4	N/A	0.09/1.51	0.08/1.13
Oil focaccia with arugula, turkey, and cheese	3986	N/A	2156/77	1830/69	N/A	5.39/19.43	5.23/19.74
Mini ciabatta with cured loin and truffle cream	3288	N/A	3288/162	N/A	N/A	8.22/40.60	N/A
Bread toast with oil, tomato, and smoked sardine	838	N/A	838/36	N/A	N/A	2.09/8.86	N/A
Toast with curry chicken	220	N/A	220/13	N/A	N/A	0.55/3.20	N/A
Shrimp skewer with garlic	1597	N/A	786/124	811/77	N/A	1.97/31.02	2.32/22.09
Spicy potatoes	4689	N/A	4689/236	N/A	N/A	11.72/59.17	N/A
Cod fritter	5780	N/A	5780/183	N/A	N/A	14.45/45.82	N/A
Falafel	5333	N/A	5333/196	N/A	N/A	13.33/49.04	N/A
Mini chicken burger	8394	N/A	7465/231	829/30	N/A	18.66/57.93	2.65/8.73
Andalusian gazpacho	1010	N/A	N/A	1010/12	N/A	N/A	2.89/3.32
Charcoal coupelle with truffled salad	176	N/A	N/A	176/13	N/A	N/A	0.50/3.80
*Esgarraet* ^d^	1319	N/A	N/A	1319/79	N/A	N/A	3.77/22.41
Piquillo pepper sandwich	803	N/A	N/A	803/22	N/A	N/A	2.29/6.34
Iberian pork sausage	146	N/A	N/A	146/15	N/A	N/A	0.42/4.22
Iberian chorizo	90	N/A	N/A	90/9	N/A	N/A	0.26/2.59
Oil and salt bread	649	N/A	N/A	649/80	N/A	N/A	1.85/22.87
Coca fine ratatouille and sausage	553	N/A	N/A	553/17	N/A	N/A	1.58/4.90
Melon	1026	N/A	1023/76	N/A	N/A	2.57/19.00	N/A
Kiwi	373	N/A	373/47	N/A	N/A	0.93/11.68	N/A
Pineapple	223	N/A	223/19	N/A	N/A	0.56/4.71	N/A
Cheesecake	4833	N/A	4833/209	N/A	N/A	12.08/52.23	N/A
Melon with lemon	2352	N/A	N/A	2352/191	N/A	N/A	6.72/54.61
Mini chocolate waffles	4022	N/A	N/A	4022/214	N/A	N/A	11.49/61.06

* Portion: Individual portions of contracted and served food at each conference, so the portion size tends to vary according to the specificities and requirements requested for each event. ^a^ Congress events; 1, 2, and 3 were celebrated on the 5 May, 25 May, and 1 June 2023, respectively. ^b^ Round-grained rice, *bajoqueta* and *tavella* (varieties of green beans), rabbit, chicken, sometimes duck, and *garrofó* (a variety of butter beans), cooked in olive oil and chicken broth. ^c^ Seafood dish similar to paella but with pasta noodles instead of rice. ^d^ Grilled red pepper salad, cured cod, garlic, olive oil, and black olives. ^e^ N/A: Not applicable. The dish was not served at that event.

**Table 4:** Magnitude of food waste grouped for food groups [56] at the three studied congress events, along with reference g/day, according to EAT-Lancet Planetary Health Diet [56].

Food Groups ^a^	Total/per Capita Amount (Grams) of Food Waste per Food Group at the Congress Event			Sum of g of Food Waste per Food Group at the Three Studied Congress Events	Mean ± SD of g of Food Waste per Food Group	Reference g/day [28]
	Event 1 ^b^ (*n* = 270)	Event 2 ^b^ (*n* = 400)	Event 3 ^b^ (*n* = 350)			
Cereals	16,795.7/62.21	15,572/38.93	19,026/54.36	51,393.7	17,131.2 ± 1751.3	232
Tubers	N/A	4689/11.72	119/0.34	4808	2404 ± 3.231.5	50
Vegetables	1490.5/5.52	2431/6.08	4362/12.46	8283.5	2761.17 ± 1463.9	300
Fruits	N/A	1628.4/4.07	2358.8/6.74	3987.2	1993.6 ± 516.5	200
Dairy	2451/9.08	5305/13.26	876/2.50	8632	2877.3 ± 2245.1	250
White meats	1412.6/5.23	594/1.48	546/1.56	2552.6	850.9 ± 487.1	29
Red meats	N/A	5851/14.63	323/0.92	6174	3087 ± 3908.9	14
Fish	965.3/3.58	8129/20.32	1853/5.29	10,947.3	3649.1 ± 3905.0	28
Eggs	N/A	N/A	N/A	N/A	N/A	13
Legumes	N/A	5333/13.33	N/A	5333	5333	75
Nuts	N/A	N/A	N/A	N/A	N/A	50
Added fats	N/A	41/0.10	N/A	41	41	51.8
Added sugars	N/A	N/A	N/A	N/A	N/A	31
Ultra-processed foods	N/A	N/A	1187/3.39	1187	1187	N/A

^a^ Food group classification according to the EAT-Lancet Planetary Health Diet [56]. ^b^ Congress events; 1, 2, and 3 were celebrated on the 5 May, 25 May, and 1 June 2023, respectively. N/A: Not applicable. The food group was not included at that event.

**Table 5:** Energy and nutritional value of food waste discarded at the three studied congress events.

	Value of Energy per Nutrient Obtained in the Food Waste/Food Group at the Congress Event			Sum of Value of Energy per Nutrient Obtained in the Food Waste/Food Group at the Three Studied Congress Events	Mean ± SD of Energy per Nutrient Obtained in the Food Waste/Food Group at the Three Studied Congress Events
	5 May 2023	25 May 2023	1 June 2023		
Energy (kcal)	42,381.04	162,330.76	81,080.77	285,792.57	95,264.19 ± 61,219.78
Protein (g)	1586.12	51.61	62.13	1699.86	566.62 ± 882.93
Carbohydrate (g)	5799.72	121.81	136.69	6058.22	2019.41 ± 3273.86
Fat (g)	2249.64	81.90	100.50	2432.04	810.68 ± 1246.21
Dietary fiber (g)	429.59	9.67	10.24	449.50	149.83 ± 242.28
Cholesterol (mg)	4549.86	276.00	329.10	5154.96	1718.32 ± 2452.33
SFA ^a^ (g)	187.84	22.82	24.78	235.44	78.48 ± 94.71
MUFA ^b^ (g)	498.67	38.00	51.25	587.92	195.97 ± 262.22
PUFA ^c^ (g)	161.02	12.44	10.16	183.62	61.21 ± 86.45
Thiamin (mg)	13.75	0.73	0.67	15.15	5.05 ± 7.53
Riboflavin (mg)	13.96	0.79	0.77	15.54	5.18 ± 7.61
Niacin (mg)	739.98	22.60	23.87	786.45	262.15 ± 413.81
Vitamin B_6_ (mg)	28.06	1.65	1.50	31.38	10.46 ± 15.24
Vitamin B_12_ (µg)	68.30	4.32	5.39	78.02	26.01 ± 36.64
Folate (µg)	4071.29	130.49	148.30	4350.08	1450.03 ± 2270.10
Vitamin C (mg)	1642.31	60.95	88.82	1792.08	597.36 ± 905.06
Vitamin A (µg)	11,579.76	321.07	294.04	12,194.87	4064.96 ± 6508.02
Vitamin D (µg)	21.92	2.52	3.33	27.77	9.26 ± 10.97
Vitamin E (mg)	129.93	7.66	6.77	144.35	48.12 ± 70.85
Vitamin K (µg)	1663.31	59.00	95.52	1817.83	605.94 ± 915.89
Calcium (mg)	9199.63	443.10	494.23	10,136.96	3378.99 ± 5040.89
Phosphorus (mg)	17,313.12	968.54	1096.30	19,377.96	6459.32 ± 9399.89
Iron (mg)	256.84	9.97	10.87	277.69	92.56 ± 142.27
Iodine (µg)	2163.53	92.36	108.77	2364.66	788.22 ± 1191.08
Magnesium (mg)	4741.81	153.51	168.30	5063.62	1687.87 ± 2644.80
Zinc (mg)	154.95	5.96	6.11	167.02	55.67 ± 85.98
Sodium (mg)	52,988.32	2759.25	3577.20	59,324.77	19,774.92 ± 28,766.55
Potassium (mg)	33,476.05	1241.30	1225.30	35,942.65	11,980.88 ± 18,615.36
Selenium (µg)	3561.57	63.03	93.88	3718.48	1239.49 ± 2011.04

^a^ SFA: Saturated fatty acid. ^b^ MUFA: Monounsaturated fatty acid. ^c^ PUFA: Polyunsaturated fatty acid.

## Data Availability

Data is contained within the article.
